# The Protective Action of *Coutarea hexandra* (Rubiaceae) on the Neuromuscular Blockade Induced by *Lachesis muta muta* (Viperidae: Crotalinae) Venom

**DOI:** 10.1155/2024/4714510

**Published:** 2024-11-15

**Authors:** Grazielle D. Pilon, Anna P. Farias-de-França, Nathalia M. Cantuária, Magali G. Silva, Aline G. Leão-Torres, Rafael S. Floriano, Marcio G. dos Santos, Nelson Jorge da Silva, Otto M. S. Gerlach, Valdir Cechinel-Filho, Yoko Oshima-Franco

**Affiliations:** ^1^Biomedicine Course, University of Sorocaba (UNISO), Sorocaba, São Paulo, Brazil; ^2^Pharmacy Course, University of Sorocaba (UNISO), Sorocaba, São Paulo, Brazil; ^3^Laboratory of Toxinology and Cardiovascular Research, Graduate Program in Health Sciences, University of Western São Paulo (UNOESTE), Presidente Prudente, São Paulo, Brazil; ^4^Graduate Program in Environmental Sciences, Tocantins Federal University (UFT), Palmas, Tocantins, Brazil; ^5^Graduate Program in Environmental Sciences and Health, School of Medical and Life Sciences, Pontifical Catholic University of Goiás (PUC Goiás), Goiânia, Goias, Brazil; ^6^Graduate Program in Pharmaceutical Sciences and Núcleo de Investigações Químico-Farmacêuticas, Universidade do Vale do Itajaí (UNIVALI), Itajaí, Santa Catarina, Brazil; ^7^Graduate Program in Pharmaceutical Sciences, University of Sorocaba (UNISO), Sorocaba, São Paulo, Brazil

## Abstract

Envenomations by snakes represent a neglected health problem in tropical and subtropical countries. In South America, *Lachesis muta* occasionally causes severe human envenomation, with treatment being conditioned to an unspecific antivenom. In this work, we examined the neutralizing ability of *Coutarea hexandra* stem bark hydroalcoholic extract (*Ch*-E), including the commercial phytochemicals coumarin and quinine, on the neuromuscular blockade induced by *L. m. muta* venom in mouse phrenic nerve-diaphragm preparation. Biological assays were performed following conventional myographic technique ex vivo. *Ch*-E was phytochemically characterized to detect the presence of coumarin and quinine using analytical methods. *Ch*-E and commercial phytochemicals were tested separately or combined under pre- and post-venom incubation protocols. *Ch*-E attenuated the venom-induced neuromuscular blockade only under the pre-venom incubation protocol. Quinine was not detected in *Ch*-E. Commercial coumarin and quinine exhibited a concentration-dependent counteracting effect on the venom-induced neuromuscular blockade. The pre-venom incubation protocol showed to be efficient in attenuating the *L. m. muta* venom–induced neuromuscular blockade, most likely due to the presence of coumarin derivatives and unknown alkaloids in this extract.

## 1. Introduction

In Brazil, envenomations by Viperidae snakes represent a critical public health problem due to the large number of cases and their severity [1]. Envenomations by *Lachesis* are frequently notified every year in the country, being exceeded only by accidents with *Bothrops* and *Crotalus* snakes [1–4]. In South America, the *Lachesis* genus is represented by a single species (=*Lachesis muta*) found in the Amazon River basin (=*L. m. muta*) and the Atlantic rainforest (=*Lachesis muta rhombeata*) [5–7]. *L. muta* venom is characterized by causing haemostatic disorders that result in gingival, ear, and eye bleeding [8–10], including local damage evidenced by myonecrosis, pain, oedema, and ecchymosis [11]; neurotoxic effects have also been reported [12, 13]. Envenomation by *Lachesis* exhibits clinical impairments similar to *Bothrops* [1, 14–17].

Envenomation by *L. muta* is conventionally treated with a polyvalent antivenom (anti-*Bothrops*/*Lachesis* serum) at an antivenom:venom ratio of 1:5 (*v*/*w*), where 1 mL of antivenom might neutralize 5 mg of *Bothrops* spp. venoms and 3 mg of *L. muta* venom, based on the similar clinical manifestations caused by these snakes in humans [16].

Alternatively, some medicinal plants had their biological properties tested against the effects of *L. muta* venom such as *Plinia jaboticaba* [18], *Lippia sidoides* [19], *Malpighia emarginata* [20], *Laurencia aldingensis* [21], *Erythroxylum* ssp. [22], *Manilkara subsericea* [23], and *Eclipta prostrata* [24], demonstrating their potential to minimize the pathophysiological occurrences induced by this venom in different experimental conditions. These studies align with the World Health Organization's policy, which encourages research into new treatments, diagnostics, and health device innovations aimed at improving treatment outcomes and hastening recovery for patients [25]. Here, we have selected *C. hexandra* (Jacq.) K. Schum (Rubiaceae), a Brazilian native plant popularly known as Quina [26], to expand the understanding of its protective action against *L. m. muta* venom.

Recently, Leão Torres et al. [27] reported that the *C. hexandra* stem bark hydroalcoholic extract (*Ch*-E) efficiently prevented systemic disorders induced by *L. m. muta* venom in rats, based on its antioxidant ability in successfully scavenging the stable radical 2,2-diphenyl-1-picrylhydrazyl [28]. *C. hexandra* exhibits other therapeutic properties such as suppressive activity of inflammatory responses, including antinociceptive and anticancer actions [29, 30]. Traditionally, *C. hexandra* has been used as a replacement extract for *Cinchona officinalis*, a South American Rubiaceae rich in quinine, which has not been tested against snake venom activities. In addition, a synthetic quinolinone inhibitor (2-hydroxymethyl-6-methoxy-1,4-dihydro-4-quinolinone) showed its efficiency in counteracting snake venom metalloprotease-induced proteolytic and hemorrhagic activities [31].

In this study, we have tested different chemical approaches to identify the presence of quinine in the *C. hexandra* stem bark hydroalcoholic extract (*Ch*-E), considering its popular use as an alternative therapeutic strategy to snakebites. In addition, we also compared the neutralizing action of *Ch*-E to commercial phytochemicals coumarin and quinine from *C. hexandra* and *Cinchona* spp., respectively, on the neuromuscular effect induced by *L. m. muta* venom ex vivo [32].

## 2. Material and Methods

### 2.1. Reagents

The *L. m. muta* venom used here was from the same pool used in a previous investigation [27] and it was obtained from one male adult snake captured in the Amazon region. The venom was desiccated and stored at −20°C until used; the venom was resuspended in ultrapure water immediately prior to the experiments. This study was registered with the Brazilian National System for the Management of Genetic Patrimony and Associated Traditional Knowledge (SISGEN, registration no. A843915). Coumarin (1,2-benzopyrone, code C4261) and quinine (6⁣′-methoxycinchonidine, code 69311) were purchased from Sigma-Aldrich Chemical Co. (St. Louis, Missouri, United States). *Ch*-E was supplied by Tocantins Federal University (Palmas, TO, Brazil) through Dr Marcio G. Santos under a 70% hydroalcoholic solution (ethanol, *v*/*v*) at a 10% concentration (*m*/*v*). *Ch*-E was lyophilized and stored at −20°C until used; *Ch*-E was resuspended in aqueous solution immediately prior to the experiments. Stem barks were collected in June/2011 from a tree georeferenced at S10°44⁣′56.4⁣^″^; W048°20⁣′08.0⁣^″^ (El. 293 m) (Porto Nacional, TO, Brazil). The *Ch*-E used here was from the same pool used in previous investigations [27]. The Exsiccate No. 9708 was deposited at the Tocantins Herbarium of Tocantins Federal University (Porto Nacional, TO, Brazil).

### 2.2. Equipment's Characterization

#### 2.2.1. Thin Layer Chromatography (TLC)

A spot of *Ch*-E was plotted first in a paper of chromatography and later in a silica gel GF254 (0.25 mm) chromatoplate (MN-Macherey-Nagel GmbH & Co. KG, Handelsgesellschaft, Germany). It was run in a mobile phase of chloroform:diethylamine (9:1) and dried in an oven at 100°C for 10 min. Two revelators were applied to the chromatoplate: Dragendorff's reagent (DR) and sulfuric acid 50% in ethanol (*w*/*v*). These revelators in contact with alkaloids produce an orange or orange-red precipitate and a blue color [33], which is visualized using UV at 365 nm [34], respectively. A positive result for quinine is an intense blue fluorescence. Sample solution: 0.5 mL of *Ch*-E solution (200 mg/mL) which was subjected to an organic extraction (4X) by a partition at alkaline pH = 10 (NaOH 0.1 M, Synth) using 5 mL dichloromethane (Synth) and 0.1 mL NH_4_OH 25% weight/volume (Synth) was taken [34]. Organic fractions were dried to completion, resuspended in 1.5 mL of absolute ethanol, and designated as purified organic extract.

#### 2.2.2. Ultraviolet Absorption Spectrophotometry

The maximum absorption of quinine occurs at 250 nm [35]. Blank solution absorbance of HCl 0.1 M was used as a zero reference [34]. This spectrophotometric profile was obtained using a UV-M51 spectrophotometer (BEL Engineering, Rio de Janeiro, RJ, Brazil).

#### 2.2.3. High-Performance Liquid Chromatography (HPLC)

Samples (extract and quinine) were prepared in methanol after 10x dilution of an aqueous *Ch*-E solution (200 mg/mL) and direct solubilization in a flask with 2 mL methanol, respectively. Both samples were filtered and injected (10 *μ*L) following the gradient: solution A (acetonitrile, ACN), solution B (methanol, MeOH), solution C (ultrapure water+0.2% formic acid), flux (1 mL.min^−1^), temperature of 40°C, Column C18 (150 × 4.6 mm, 5 *μ*m). They were collected at 360 nm based on the UV profile of quinine, which has a spectral distribution from 320 to 400 nm and a maximum of 362 nm [36].

### 2.3. Animals

Male Swiss mice (25–30 g) were purchased from Anilab (Laboratory Animals, Paulínia, SP, Brazil). The animals were housed at 25 ± 3°C in a light/dark cycle of 12 h and had free access to food and water ad libitum. This study was approved by the Animal Ethics Committee of University of Sorocaba (protocol no. 165/2019), and the experiments were performed according to the international guideline—ARRIVE [37].

### 2.4. Tests on Animal Models

The diaphragm and both phrenic nerves (mouse phrenic nerve-diaphragm preparation, PND) were obtained from mice anaesthetized with isoflurane (Instituto Biochimico Ltda, Itatiaia, RJ, Brazil) and sacrificed by exsanguination. Each hemidiaphragm was mounted under a tension of 0.5 g in a 5 mL organ bath [38] containing Tyrode solution aerated with carbogen (95% O_2_ and 5% CO_2_) and maintained at 37°C. The Tyrode solution maintains the physiological conditions of the neuromuscular preparation at pH 7.0 and consists of (in mM) NaCl 137, KCl 2.7, CaCl_2_ 1.8, MgCl_2_ 0.49, NaH_2_PO_4_ 0.42, NaHCO_3_ 11.9, and glucose 11.1. The preparation was indirectly stimulated through the phrenic nerve (ESF-15D double physiological stimulator) using supramaximal stimuli under a frequency of 0.06 Hz and duration of 0.2 ms. The isometric twitch tension was recorded with a force-displacement transducer (cat. 7003, Ugo Basile, Italy) coupled to a digital recorder system (Data Capsule, cat. 17400, Ugo Basile) containing a basic preamplifier (cat. 7080, Ugo Basile), coupled to a computer via a USB interface for data aquisition. After recording under controlled conditions during the stabilization of the preparation for 10 min, the pharmacological protocols were performed using different concentrations of *Ch*-E added into the bath (100, 200, and 1000 *μ*g/mL) (*n* = 6 each) [39]. The minor concentration of *Ch*-E was selected to be used in the neutralization assays, considering the concentration of 100 *μ*g of venom/mL [40]. Since such a plant expresses coumarin derivatives and quinine could be present in *Ch*-E, as it is present in *Cinchona* spp., the neutralizing action of these phytochemicals (Sigma-Aldrich) was also examined against *L. m. muta* venom. In addition, we have applied two protocols to assess the neutralizing ability of *Ch*-E in PND preparations exposed to *L. m. muta* venom: (1) pre-venom incubation: *Ch*-E and venom were pre-incubated for 30 min at room temperature before exposing the mixture to preparation; (2) postvenom: preparations were previously stabilized and subjected to the venom for 10 min, and then *Ch*-E was directly added into the bath, with responses being continuously recorded up to 120 min. The contractile efficiency was analyzed in all ex vivo protocols. This approach is suitable for contraction protocols in which the muscle length changes cyclically. It is mandatory to ensure that the length and force output are the same at the start and end of the measurement period. Thus, the contractile efficiency was analyzed throughout the 120-min experimental period [41, 42].

### 2.5. Data Analysis

The results were expressed as the mean ± standard error of the mean (SEM) and were compared among groups using one-way ANOVA followed by the Tukey test, with *p* < 0.05 indicating significance in all cases. Data analyses were performed using Origin v.9.5 (OriginLab Corporation, Northampton, MA, United States).

## 3. Results

### 3.1. *C. hexandra* Stem Bark Hydroalcoholic Extract (*Ch*-E) Chemical Characterization

**Figure 1:**
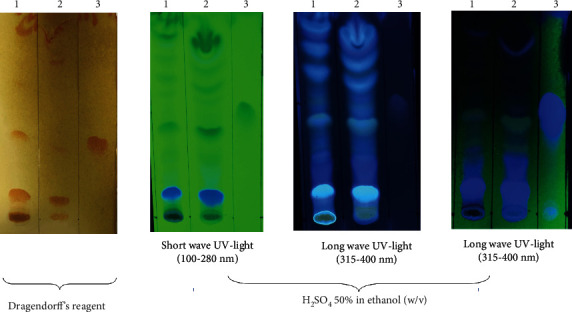
Thin layer chromatography on silica gel GF254 (0.250 nm). Mobile phase: CHCl_3_: (C_2_H_5_)_2_NH (90:10). (1) *Coutarea hexandra* extract. (2) Purified organic extract. (3) Quinine.

Chemical tests to detect quinine in *Ch*-E were performed using TLC, spectrophotometry, and HPLC. In TLC, the investigation was conducted by nebulizing chromatoplates with the DR or H_2_SO_4_ 50% in ethanol (*w*/*v*) in order to generate an orange color or blue fluoresce, respectively, for positive results for alkaloids, including quinine (Figure 1).

Here, the presence of alkaloids is unequivocal, as selectively shown using the DR. The chromatoplate nebulized with H_2_SO_4_ 50% in ethanol shows the complexity of the chromatographical profile of *Ch*-E. It is possible to notice the intense blue fluorescence at short-wave UV light, which is different from the quinine pattern, or even at long-wave UV light. Although the experimental condition is specific to alkaloids, these results do not yet confirm the presence of quinine in *Ch*-E.

**Figure 2:**
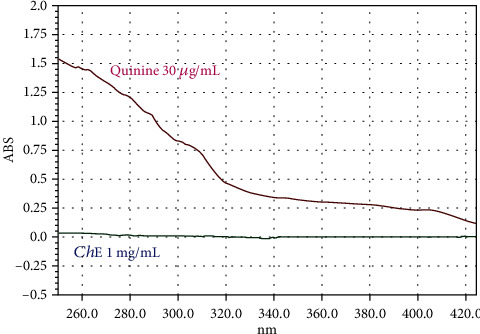
Comparative analysis of the UV absorption spectra of quinine (30 *μ*g/mL) and *Ch*-E (1 mg/mL) showing the lack of quinine. Note the maximum spectrum of quinine in an acid medium in ~250 nm.

In addition, *Ch*-E (1 mg/mL) was spectrophotometrically compared to quinine (30 *μ*g/mL), as shown in Figure 2.

**Figure 3:**
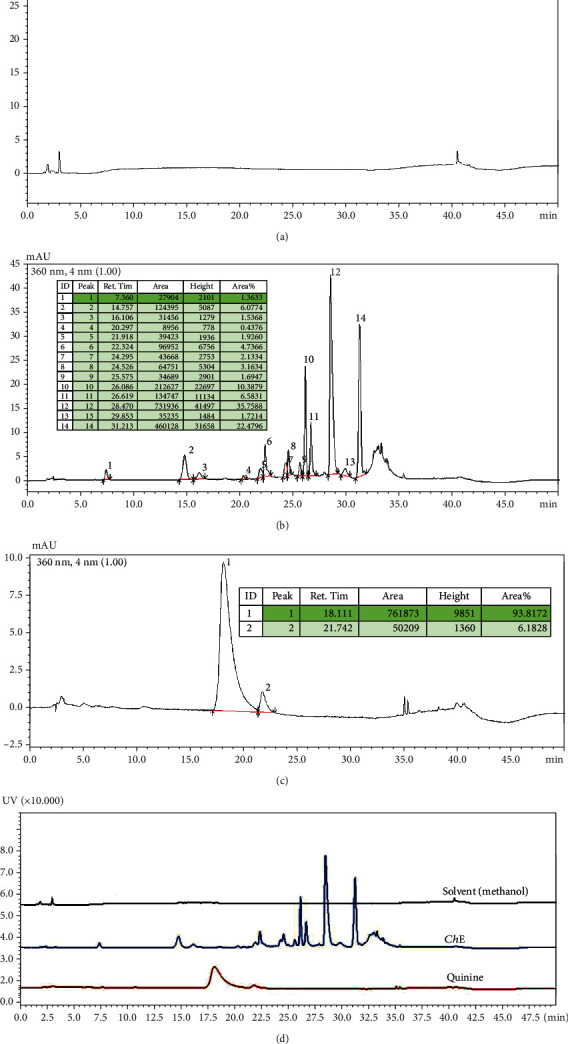
Chromatographic profile of (a) methanol solvent, (b) *Ch*-E, and (c) quinine via HPLC. (d) Comparison among the three analyses, showing the different chromatographic profiles of quinine in comparison to the *Ch*-E.

Due to the absence of quinine in *Ch*-E from the UV spectrophotometry, a new set of experiments was conducted using an improved analytical technique. The results are presented in Figure 3, where Figure 3(a) represents the methanol solvent, Figure 3(b) displays the *Ch*-E chromatogram with several peaks, along with the corresponding parameters shown in the inserted table, Figure 3(c) shows the quinine chromatogram with two peaks, also detailed in the table, and Figure 3(d) illustrates the chromatogram combining the three analyses, conclusively confirming the absence of quinine in the *Ch*-E.

### 3.2. Concentration-Response Curve of *Ch*-E in Isolated Preparation

**Figure 4:**
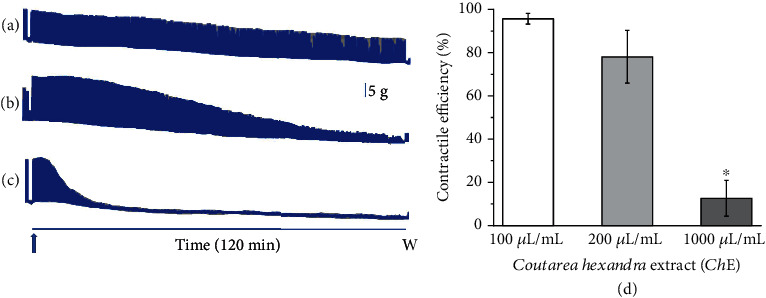
Representative recordings of the concentration-response test of *Ch*-E ((a) 100, (b) 200, and (c) 1000 *μ*L/mL) in indirectly stimulated mouse PND preparation (*n* = 5 each). Bar: tension (5 g/cm); arrow: the moment *Ch*-E was added into the bath; W: Tyrode solution washing-out. (d) Contractile efficiency (in percent) after 2 h of the experiment confirmed the best response of 100 *μ*L/mL. ⁣^∗^*p* < 0.05 in comparison to 100 and 200 *μ*L/mL concentration of *Ch*-E.

The effect of *Ch*-E was assayed in mouse neuromuscular preparations. Ideally, the best concentration for counteracting venom paralysis is the one that does not cause changes in the basal response, as shown by Figure 4(a) (100 *μ*g/mL), in opposite to 200 or 1000 *μ*g/mL (Figures 4(b) and 4(c), respectively). The contractile efficiency (in percent) under *Ch*-E exposure (Figure 4(d) was 95.7 ± 2.5, 78.1 ± 12.2, and 12.6 ± 8.3 for 100, 200, and 1000 *μ*g/mL, respectively.

### 3.3. Counteracting the Venom-Induced Paralysis Under Pre- and Post-venom Incubation Protocols

**Figure 5:**
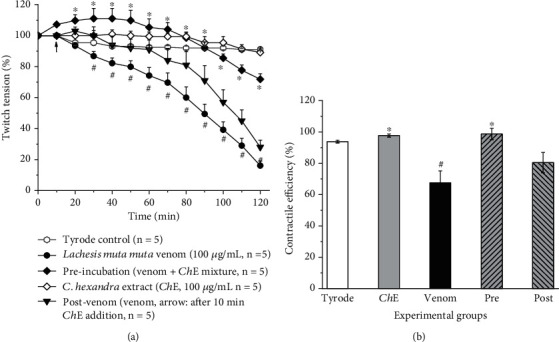
Neutralizing assay with *C. hexandra* extract (*Ch*-E) on the neuromuscular blockade induced by *L. m. muta* venom in indirectly stimulated mouse PND preparation. (a) *Ch*-E (♢), whose profile is like control (〇) prevented the venom-induced neuromuscular blockade (●) only by applying the preincubation protocol (♦), but not the postvenom protocol (▼). (b) Contractile efficiency after 2 h of experiment. In (a), the points are the mean ± SEM, *n* = 5. ^#^*p* < 0.05 compared to control (Tyrode solution) and *Ch*-E. ⁣^∗^*p* < 0.05 compared to venom. Arrow indicates the addition of *Ch*-E after 10 min. Pre, preincubation protocol; post, postincubation protocol.

In this set of experiments (Figure 5), a concentration of 100 *μ*g of *L. m. muta* venom/mL was selected. The ability of the venom to inhibit the muscle contractility is distinguishable. Aiming at counteracting this toxic effect, two protocols were carried out (pre- and post-venom incubation).

In Figure 5(a), the outcome of the pre-venom incubation protocol clearly shows an interaction between components of *C. hexandra* and constituents of the venom because when the mixture was added into the bath, the effect was a slight facilitation (an increase in twitches amplitude), which is not expressed by the venom alone, nor by *Ch*-E alone. Besides, the characteristic venom blockade was significantly avoided (⁣^∗^*p* < 0.05). However, when the venom was directly added into the bath, it triggered the toxic paralysis, which was not avoided by *Ch*-E, even when added early (10 min).

To help in the interpretation, we measured the contractile efficiency (Figure 5(b)). The muscular work was as follows (in percent): Tyrode 93.8 ± 0.8; *Ch*-E, 97.7 ± 1; venom 67.6 ± 7.5; pre-venom incubation protocol, 98.7 ± 3.6; and post-venom incubation protocol, 80.4 ± 6.5. This analysis provides insights into the muscle's effectiveness in generating work under various conditions, offering a perspective on the impact of different factors such as treatments or interventions on its contractile performance. Taking these results together and considering the presence of a myriad of components in the venom, it is suggestive to think that the venom triggers its paralysis mechanism before myotoxic events, which *Ch*-E neutralized in the pre-venom incubation protocol, but not when the venom started its neurotoxic activity (post-venom incubation protocol).

Thus, based on coumarin derivatives found in *C. hexandra* and searching by the effect of quinine (not yet studied against the neuromuscular blockade induced by *L. m. muta* venom) as representative of *Cinchona* spp., a sequence of different amounts of these commercial phytochemicals were assayed.

### 3.4. Counteracting the Venom-Induced Paralysis Under Pre-Venom Incubation Protocol Using Coumarin

**Figure 6:**
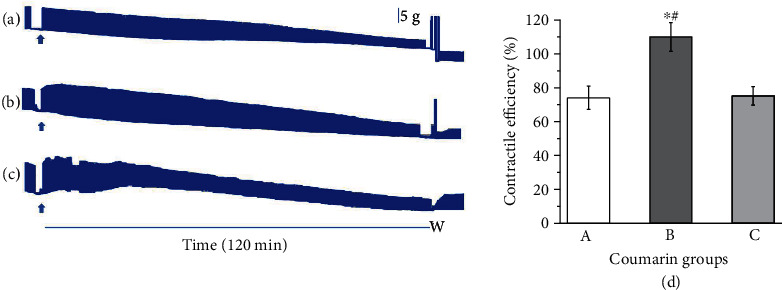
Mouse phrenic nerve-diaphragm preparation under indirect stimulation. Representative myographic recordings showing (a) preincubation protocol (1:1) of *L. m. muta* venom (100 *μ*g/mL)+coumarin (100 *μ*g/mL), (b) preincubation protocol (1:2) of *L. m. muta* venom (100 *μ*g/mL)+coumarin (200 *μ*g/mL), and (c) coumarin (200 *μ*g/mL) alone. Arrows indicate the addition of mixture into the bath. W: Tyrode solution washing-out. Bar = tension (5 g/cm) in all experiments. (d) Contractile efficiency (in percent) after 2 h of experiment of the same groups (A, B, and C) confirmed the better response of 100 *μ*L/mL. ⁣^∗^*p* < 0.05 in comparison to 100 and 200 *μ*L/mL concentration of *Ch*-E.

Figure 6 shows a set of experiments carried out only using commercial coumarin as representative of coumarin derivatives in plants. When pre-incubated with the venom, both the proportion of coumarin outlined in Protocol A (1:1, Figure 6(a)) and double that amount as per Protocol B (1:2, Figure 6(b)) demonstrated that a higher concentration of this phytochemical, as depicted in Protocol C (coumarin 200 *μ*g/mL, Figure 6(c)), corresponded to increased contractile efficiency (Figure 6(d)). The contractile efficiency (in percent) was 74.1 ± 6.8, 110 ± 8.5, and 75.2 ± 5.5 for these groups (A, B, and C).

### 3.5. Counteracting the Venom-Induced Paralysis Under Pre-Venom Incubation Protocol Using Quinine

**Figure 7:**
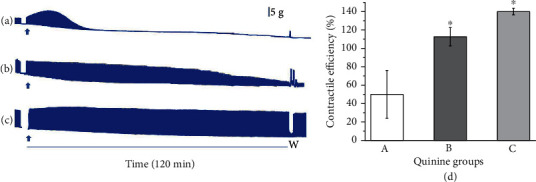
Mouse phrenic nerve-diaphragm preparation under indirect stimulation. Representative myographic recordings showing (a) preincubation protocol (1:1) of quinine (100 *μ*g/mL)+*L. m. muta* venom (100 *μ*g/mL), (b) preincubation protocol (1:5) of quinine (20 *μ*g/mL)+*L. m. muta* venom (100 *μ*g/mL), and (c) quinine (20 *μ*g/mL) alone. W: Tyrode solution washing-out. Bar = tension (5 g/cm) in all experiments. Arrows indicate the addition of mixture into the bath. (d) Contractile efficiency after 2 h of experiment of the same groups (A, B, and C). ⁣^∗^*p* < 0.05 compared to A.

Figure 7 shows a set of experiments carried out only using commercial quinine combined with the venom (1:1, Figure 7(a); 1:5, Figure 7(b)) or quinine (20 *μ*g/mL; Figure 7(c)) alone, as representative of quinine in *Cinchona* spp.

The analyzed contractile efficiency (Figure 7(d)) considering the experimental period showed that the pre-incubation in A (1:1, 50 ± 26%) was statistically different from the pre-incubation in B (112.6 ± 10%) and quinine alone (C, 140 ± 3.6%), with no statistical difference between B and C, showing a positive trend towards decreasing quinine in pre-incubation.

### 3.6. Counteracting the Venom-Induced Paralysis Under Pre-Venom Incubation Protocol Using Coumarin and Quinine Simultaneously

**Figure 8:**
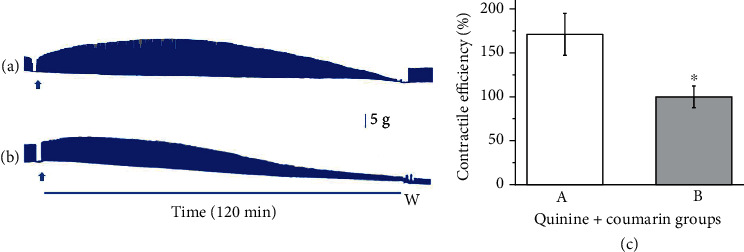
Mouse phrenic nerve-diaphragm preparation under indirect stimulation. Representative myographic recordings showing (a) preincubation protocol (2:1:1) of *L. m. muta* venom (100 *μ*g/mL)+quinine (50 *μ*g/mL)+coumarin (50 *μ*g/mL) and (b) preincubation protocol (5:1:9) of *L. m. muta* venom (100 *μ*g/mL)+quinine (20 *μ*g/mL)+coumarin (180 *μ*g/mL). Arrows indicate the addition of mixture into the bath. W: Tyrode solution washing-out. Bar = tension (5 g/cm) in all experiments. (c) Contractile efficiency after 2 h of experiment of the same groups (A and B). ⁣^∗^*p* < 0.05 compared to A.

The role of coumarin and quinine was explored in this study as representative phytochemicals of *C. hexandra* and *Cinchona* spp., respectively, against the neuromuscular blockade induced by *L. m. muta* venom. Due to the trends pointed out with coumarin (200 *μ*g/mL, Figure 6(d)) and quinine (20 *μ*g/mL, Figure 7(c)), a set of experiments was carried out.  Figure 8 shows the pre-incubation with venom and coumarin+quinine simultaneously. A synergic effect between coumarin and quinine was investigated in the first protocol (Figure 8(a)). Notice the twitches' recovery after the Tyrode solution washing-out. In Protocol B (Figure 8(b), the best responses shown in previous experiments with quinine (20 *μ*g/mL) and coumarin (200 *μ*g/mL) were assayed, discounting 20 of quinine and reducing coumarin to 180, which sum gives 200 *μ*g/mL. The contractile efficiency shows a synergic effect with equimolar concentrations of quinine and coumarin (Figure 8(c) and a good response in Protocol B.

When the contractile efficiency was analyzed throughout the experimental period, the same amount of quinine and coumarin was the better response (A, 171 ± 23.9%) in comparison to the decreased quinine and increased coumarin (B, 100.9 ± 12.3%), as pointed out in isolate experiments or with quinine or with coumarin. Even in conditions that culminate in paralysis, it is possible to measure the work performed by muscle cells due to their safety margin.

## 4. Discussion

In this study, we have characterized *Ch*-E, known as “quina” (or china), used in Brazil as a substitute for *Cinchona* spp., focusing on alkaloids since coumarin derivatives from *C. hexandra* have already been identified such as 5-hydroxy-7-methoxy-4-(2,5-dihydroxy phenyl)-2H-1-benzopyran-2-one [43], 8-hydroxy-5,7,3⁣′,4⁣′-tetramethoxy-4-phenylcoumarin [44], and 4-arylcoumarins [45], including two coumarins isolated by Olmedo et al. [30] (5-O-beta-D-glucopyranosyl-4-(4-hydroxyphenyl)-7-methoxy-2H-chromen-2-one and 5-O-beta-D-galactopyranosyl-4-(4-hydroxyphenyl)-7-methoxy-2H-chromen-2-one), as well as three cucurbitacins (23,24-dihydro cucurbitacin F, 23,24-dihydro-25-acetylcucurbitacin F, and 2-O-beta-D-glucopyranosyl-23,24-dihydrocucurbitacin F).

Phytochemical screening is a straightforward, uncomplicated, and cost-effective technique. It offers quick insights regarding the numerous phytochemicals present in a mixture. This method holds significance in the analysis of bioactive compounds, serving as an essential tool in identifying and studying such compounds [46]. The DR identifies alkaloids, which contain one or more nitrogen atoms, usually in combination as part of a cyclic system. These compounds are characterized by their bitter taste, which can lead to confounding with species rich in quinine, as found in *Cinchona officinalis* [35]. TLC (Figure 1) remains popular due to its convenient setup, cost-effectiveness, and accessibility to diverse stationary phases. Silica, alumina, cellulose, and polyamide are particularly effective in separating phytochemicals. However, the complex nature of phytochemicals in plant materials poses challenges for separation. Despite this, TLC's ability to employ multiple mobile phases to increase polarity aids in achieving valuable separations [47].

The use of UV-Vis spectrophotometry increased the confidence regarding the absence of quinine in *Ch*-E since the method allowed for the quantification of compounds [48]. UV absorbance measurements of quinine (30 *μ*g/mL) brought the spectrum to a wavelength range of ~250 nm (Figure 2), but not in *Ch*-E (1 mg/mL), showing the absence of quinine in *Ch*-E. Then, HPLC, a widely used analytical technique, was used to confirm these findings (Figure 3). It involves passing a sample mixture through a tightly packed column filled with solid particles, interacting with the sample compounds and a solvent in the mobile phase. This interaction allows for compound separation. HPLC is particularly well-suited for natural product isolation, offering flexibility and stability in the process [49]. HPLC is gaining popularity among various analytical methods due to its applicability to compounds with high molecular weights, limited volatility, thermal instability, and robustness. This technique has three main applications: detection, chemical separation, and purification of drugs. It is utilized across drug discovery, production, and manufacturing phases. HPLC's distinctive role in the methodological drug analysis development is a notable feature of its application [50]. Our results clearly showed the different chromatographic patterns expressed by commercial quinine and *Ch*-E.

Concerning the neuromuscular blockade induced by *L. m. muta* venom (Figure 5), Damico et al. [40] attributed neurotoxicity as an event of major significance to myotoxicity according to the results obtained using mouse phrenic nerve-diaphragm and chick biventer cervicis preparations. In a further study, using in vitro protocols, Damico et al. [51] studied the cytotoxicity of the phospholipase A_2_ (PLA_2_) isoform LmTX-I. The authors concluded that the LmTX-I did not contribute per se to the direct venom cytotoxicity, which would be mediated by metalloproteinases/disintegrins and other components of the venom. After selecting the best concentration of *C. hexandra* (Figure 4), for further assays with venom, the pre-incubation between *Ch*-E and venom was the best outcome (Figure 5). The experimental strategy of pre-incubating venoms with potential inhibitory agents (drugs, molecules, and/or plant extracts) has been widely used to test the viability of these compounds to attenuate snake venom-induced toxic effects [52–54]. Therefore, in theory, only the remaining constituents of the venom not fully neutralized by *Ch*-E would be available to induce paralysis. On the other hand, the postvenom protocol simulates the snake accident [55, 56], since the intervention is made after the snakebite.

The different responses in both protocols using the same ex vivo preparation can reflect the presynaptic nature of *Lachesis m. muta* described by Damico et al. [40], and a total disability of *C. hexandra* in avoiding the blockade progression once this action is triggered long before other venom components can act. The contractile efficiency concept, during the experimental period of 120 min, shows the success of *C. hexandra* in the pre-incubation protocol. The neuromuscular junction operates through pre- and postsynaptic events characterized by acetylcholine (ACh) vesicle release and activation of postsynaptic nicotinic receptors, respectively, which promote a muscle membrane depolarization due to a large sodium influx increasing endoplasmic calcium release and muscle contraction [57].

Although a total of 151 peptides were identified in the venom of *L. muta*, including 69 from a metalloproteinase, 58 from the BPP-CNO precursor, and 24 from an L-amino acid oxidase [58], none of these fractions were tested in ex vivo preparations to attribute the components responsible for the neuromuscular blockade. Thus, the mechanism by which *L. m. muta* induces paralysis, as shown here and elsewhere [40] in ex vivo preparations, remains to be clear.

Concerning coumarin, it is known that its neuroprotective properties are attributed to its unique oxa-heterocyclic loop structure, which enables it to bind with a wide range of proteins [59], which would include snake venoms. Due to the contractile efficiency of 200 *μ*g/mL coumarin in 75.2 ± 5.5% (Figure 6), a minor concentration of coumarin alone would be used. Previously, Buleandra et al. [60] showed that 100 *μ*g/mL coumarin does not change the basal response, which would be indicative of testing. This trial was discouraged for two reasons: the above interpretation that an increase of coumarin would provide a better outcome and previous assays using 100 *μ*g/mL coumarin addressed to *Crotalus durissus terrificus* or *Bothrops jararacussu* venom were not able to hinder the paralysis of these two snake venoms using the same neuromuscular preparation [60]. It is important to highlight the similarities of toxic effects induced by venom components of *Bothrops* and *Lachesis* species [16].

Concerning quinine, this alkaloid is one of the bitterest substances known and is significantly bitter at a molar concentration of 1 × 10^−5^ [35]. In parallel to this, it is possible to note a slight and maintained facilitation during all periods of the experiment by the low concentration of quinine (20 *μ*g/mL), reinforcing its high function as a tonic in low concentration (Figure 7). The addition of tonic water for its bitterness is a common practice, and this application has become a part of culinary and beverage traditions in many parts of the world [61, 62]. Besides, quinine in low concentration is used as a treatment for nocturnal leg cramps [63]. These last authors found that quinine blocks ACh-evoked currents, independently of the ACh concentration, in a noncompetitive manner. When in a mixture with the venom, this low concentration of quinine retarded the neuromuscular blockade. Taken together the findings indicating that quinine is effective at the muscular nicotinic ACh receptors (nAChRs) [63], and our findings, we can suggest that the quinine protection against *L. m. muta* venom could be based on targeting nAChRs. Investigating the phytochemical variation between coumarin and quinine to counteract the paralysis of *L. m. muta* venom is a captivating task that would give rise to numerous trials. For now, this study has already provided important information that can be summarized: *Ch*-E has coumarin derivatives; alkaloids, which were not identified in this study; the plant is quinine-free; it can counteract the neuromuscular blockade induced by *L. m. muta* venom in the pre-venom incubation protocol. Phytochemical studies of this plant will allow the identification of molecules responsible for attenuating the venom's toxic properties. Such molecules might be used as an alternative therapeutic strategy to antivenom. Besides, coumarin in *Ch*-E and quinine in *Cinchona* spp. are potential phytochemicals for this purpose.

## 5. Conclusions

*Ch*-E efficiently counteracted the neuromuscular blockade induced by *Lachesis m. muta* venom in PND preparation. *Ch*-E contains compounds that might attenuate the toxic effect of this venom, as evidenced when the venom and extract were pre-incubated together. Some phytochemicals may be involved in this response, such as coumarin derivatives and alkaloids, but quinine was not identified in this extract. Besides, experiments carried out using commercial coumarin and quinine revealed concentration-dependent action on the neuromuscular paralysis induced by *L. m. muta* venom. These findings may contribute to developing new antivenom therapeutic strategies as a complement to commercial antivenom or as its replacement in remote areas.

## Data Availability

The research data were approved by FAPESP. Data were also divulged at https://www.simposioiiqf.com.br/2022/.
